# Role of PVDF in Rheology and Microstructure of NCM Cathode Slurries for Lithium-Ion Battery

**DOI:** 10.3390/ma13204544

**Published:** 2020-10-13

**Authors:** Sang Hoon Sung, Sunhyung Kim, Jeong Hoon Park, Jun Dong Park, Kyung Hyun Ahn

**Affiliations:** 1 School of Chemical and Biological Engineering, Institute of Chemical Processes, Seoul National University, Seoul 08826, Korea; ssung@snu.ac.kr (S.H.S.); parkjh0109@snu.ac.kr (J.H.P.); 2 Institute for Mechanical Process Engineering and Mechanics, Karlsruhe Institute of Technology, Gotthard-Franz-Straße 3, 76131 Karlsruhe, Germany; sunhkim@lgchem.com; 3 Department of Chemical and Biological Engineering, Sookmyung Women’s University, Cheongpa-ro 47-gil 100, Yongsan-gu, Seoul 04310, Korea; jdpark@sookmyung.ac.kr

**Keywords:** NCM, cathode slurry, PVDF, rheology, microstructure

## Abstract

A binder plays a critical role in dispersion of coating liquids and the quality of coating. Poly(vinylidene fluoride) (PVDF) is widely used as a binder in cathode slurries; however, its role as a binder is still under debate. In this paper, we study the role of PVDF on the rheology of cathode battery slurries consisting of Li(Ni_1/3_Mn_1/3_Co_1/3_)O_2_ (NCM), carbon black (CB) and N-methyl-2-pyrrolidone (NMP). Rheology and microstructure of cathode slurries are systemically investigated with three model suspensions: CB/PVDF/NMP, NCM/PVDF/NMP and NCM/CB/PVDF/NMP. To highlight the role of PVDF in cathode slurries, we prepare the same model suspensions by replacing PVDF with PVP, and we compare the role of PVDF to PVP in the suspension rheology. We find that PVDF adsorbs neither onto NCM nor CB surface, which can be attributed to its poor affinity to NCM and CB. Rheological measurements suggest that PVDF mainly increases matrix viscosity in the suspension without affecting the microstructure formed by CB and NCM particles. In contrast to PVDF, PVP stabilizes the structure of CB and NCM in the model suspensions, as it is adsorbed on the CB surface. This study will provide a useful insight to fundamentally understand the rheology of cathode slurries.

## 1. Introduction

Rechargeable lithium-ion batteries (LIBs) are widely used in daily applications such as cordless-home-appliances and electric vehicles because of their high specific energy, light weight, and long cycle-life [1]. A typical electrode fabrication starts with preparation of battery slurry by mixing active materials, conductive agents, polymeric binders, and solvents. When the slurry satisfies appropriate viscosity, it is casted onto metal foil (Al or Cu) and dried to form porous electrodes. The dried electrodes are calendared to improve electrical conductivity and further dried to remove the residual solvent and moisture before making the cells [2]. 

Many commercial LIBs use Poly (vinylidene fluoride) (PVDF) as a binder in the cathode because of its excellent electrochemical stability, good wettability with electrolyte and acceptable binding ability between active materials and current collectors [3]. Poly (vinylidene fluoride) (PVDF) is a linear type synthetic semi-crystalline homopolymer with the repeat unit (CH2CF2). PVDF is well soluble in aprotic solvent such as Dimethylformamide (DMF), Dimethyl sulfoxide (DMSO) and N-Methyl-2-pyrrolidone (NMP), among which NMP is mainly utilized in commercial cathode LIBs.

The dispersion quality of active materials and conductive agents in slurries determines their rheological properties and critically influences the processability of a battery electrode [4,5]. Dispersion quality also affects the mechanical strength and electrical conductivity of the electrode, resulting in electrical performance of LIBs [6,7,8]. In the field of colloidal science and relevant applications, dispersion quality of particle-polymer mixture can be controlled by the interaction between particle and binder [9,10,11]. In LIBs, it has also been argued that the internal structure of the slurry is determined by the interaction between the particles and polymeric binders [12,13,14]. In the anode slurry of LIBs, it is reported that carboxymethyl cellulose (CMC) improves the dispersion quality of anode slurry as it is adsorbed on the surface of active materials(graphite) and conductive agent (carbon black), thus resulting in steric stabilization [15,16,17].

It has been reported that the aggregation of carbon black (CB) which is used as a conductive agent causes practical problems in the processing of electrode slurries [12,18]. Therefore, understanding the rheological properties originated from CB aggregates is important from both the academic and industrial points of view. In attempting to elucidate these rheological responses, extensive studies have been carried out for the CB suspensions. Because of strong van der Waals attraction and high specific surface area, CBs readily form a network structure in suspension; therefore, the occurrence of both yield stress and thixotropy for these systems has been found [19,20,21,22,23]. Polymers are mainly introduced to disperse CB as they are adsorbed on the CB surface [24,25]. 

Despite the widespread use of PVDF and its importance in the cathode, our understanding of the role of PVDF in microstructure and rheological property of cathode slurry is still lacking. Song and coworkers [26,27,28] described the PVDF as a typical adsorbing polymer in cathode slurry that plays a role in controlling dispersion quality and corresponding rheological properties. They argued that high molecular weight PVDF forms a gel structure by bridging flocculation; thus, the floc structure of the particles is affected by interaction of the particle and the polymeric binders. 

To prove the previous arguments and firmly understand the role of PVDF on the rheology of cathode slurries, systemic studies should be performed with model systems consisting of solely target components. The present study aims to reveal the role of PVDF in microstructure and rheological properties of cathode slurries consisting of Li(Ni_1/3_Mn_1/3_Co_1/3_)O_2_ (NCM) [29,30] as an active material, carbon black (CB) as a conductive agent, PVDF as a polymeric binder, and NMP as a solvent. Following the three cases of model suspensions were prepared to understand the role of PVDF in the rheology and microstructure of cathode slurries: CB/PVDF/NMP, NCM/PVDF/NMP, and NCM/CB/PVDF/NMP. To further understand the role of PVDF in cathode slurries, we prepared the same model suspensions by replacing PVDF with Polyvinylpyrrolidone (PVP), and compared the role of PVDF to PVP in the suspension rheology. 

## 2. Materials and Methods 

Li(Ni_1/3_Mn_1/3_Co_1/3_)O_2_ (NCM, Gelon, Qingdao, China) with a 10 µm of mean particle size and 4.7 g/cm^3^ of density is used as an active material as received. Carbon black (CB, Super-C65, Timcal Ltd., Bodio, Switzerland) with 62 m^2^/g of BET surface area, 35 nm mean particle diameter, 2.25 g/cm^3^ of density according to the supplier were used as a conductive agent. Poly (vinylidene fluoride) (PVDF, Solef 6020, Solvay, Brussels, Belgium) with 700,000 g/mol of molecular weight, according to the supplier, is used as a binder. Polyvinylpyrrolidone (hereafter PVP, Sigma-Aldrich, St. Louis, MO, USA) with 360,000 g/mol of molecular weight, according to the provider, are used as received. N-Methyl-2-pyrrolidone (NMP, Daejung, Siheung, Korea) is used as a solvent. 

We prepared the following three model suspensions which contain CB-only (CB/PVDF/NMP), NCM-only (NCM/PVDF/NMP), and both NCM and CB (NCM/CB/PVDF/NMP), respectively in PVDF/NMP solution. We prepared a 5 wt% PVDF/NMP stock solution by dissolving PVDF in NMP for 6 h with magnetic stirring at 60 °C To prepare CB/PVDF/NMP suspension, CB powder was added to the PVDF/NMP solution and homogenized at 8000 rpm for 3 min using a rotor-stator homogenizer (Ultra-Turrax T18, IKA, Staufen im Breisgau, Germany). CB concentration was determined to be 3 wt% (=1.3 vol%). To prepare NCM/PVDF/NMP suspension, NCM powder was added to the PVDF/NMP solution and mixed using an anchor-type overhead stirrer at 1000 rpm for 15 min. The NCM/CB/PVDF/NMP mixture was prepared by adding the NCM powder to the CB/PVDF/NMP mixture and mixing them using an anchor-type overhead stirrer at 1000 rpm for 15 min. The procedure to prepare model suspensions which replaced PVDF to PVP was the same as the PVDF-based suspensions described above. Volume fraction of the particle was calculated based on the density of the materials provided by the manufacturers.

We measured the adsorbed amount (Γ) of PVDF (or PVP) on the surface of CB and NCM, respectively, in CB/PVDF (or PVP)/NMP and NCM/PVDF (or PVP)/NMP suspensions. Γ was determined by quantifying the polymer concentration in the matrix before and after polymer adsorption. For the measurement of Γ on NCM, the suspensions containing NCM of 35 wt% and PVDF (or PVP) of 0.64 wt% were prepared. They were centrifuged at 10,000 rpm for 10 h, and 5 h more consecutively. For the measurement of Γ on CB, the suspensions containing CB of 3.23 wt% and PVDF (or PVP) of 0.97 wt% were prepared. They were centrifuged at 15,000 rpm for 10 h, and repeated three times to obtain clear supernatant. When all the particles are settled, the supernatant phase becomes transparent. The concentration of residual polymer remaining in the supernatant solution was obtained by the gravimetric analysis. The solvent was evaporated by drying in a convection oven at 110 °C, and the residue was weighed with a microbalance. The total amount of polymers adsorbed on the particle was obtained by calculating the difference between the amount of polymer in the initial solution and the supernatant solution after centrifuge. 

Rheological properties were measured in the strain-controlled mode of AR-G2 rheometer (TA Instruments, New Castle, DE, USA) using 40 mm serrated parallel plate geometry to reduce wall-slip. Loading gap was adjusted in the range from 600 µm to 800 µm depending on the initial loading amount of the sample. Steady-state viscosities were measured in the descending mode from $\dot{\gamma }$ = 100 s^−1^ to 0.01 s^−1^ using varied equilibration time from 10 s at the highest shear rate to 60 s at the lowest shear rate. Frequency sweep tests were carried out in the descending mode from ω = 100 rad/s to 0.4 rad/s within the linear viscoelastic regime for each sample. To obtain a reproducibility of the frequency sweep test, a pre-shear protocol of 20 s^−1^ for 10 s and a rest time for 10 s were applied.

Optical microscope images were obtained using a polarizing microscope (BX51, Olympus, Tokyo, Japan). Suspensions were diluted enough to be observable with microscope. A volume of 0.1 mL of the suspension was loaded onto a slide glass measuring 24 × 50 mm^2^ and covered with a cover glass measuring 22 × 22 mm^2^. 

## 3. Results and Discussion

### 3.1. CB/Polymer/NMP Suspension

**Figure 1:**
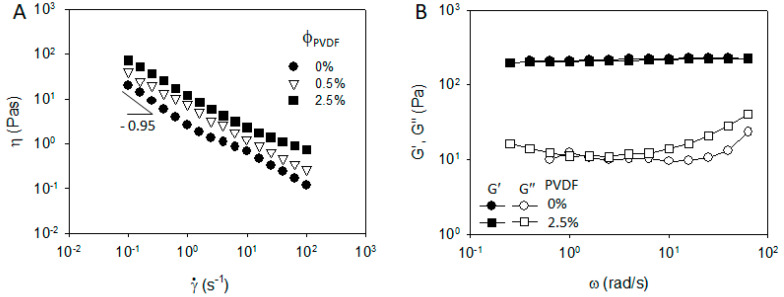
(**A**) Steady shear viscosity and (**B**) frequency dependent storage and loss modulus of carbon black (CB)/Poly(vinylidene fluoride) (PVDF)/N-Methyl-2-pyrrolidone (NMP) mixtures of 3 wt% (=1.3 vol%) of CB with a varying polymer concentration.

In this section, the rheological properties and optical microscope images of CB/PVDF/NMP and CB/PVP/NMP suspensions are compared. Steady shear viscosity (η) and frequency-dependent viscoelastic moduli (G′ and G″) of CB/PVDF/NMP were measured at various polymer concentrations, as shown in Figure 1. The viscosity in the absence of PVDF (i.e, ϕ_PVDF_ = 0 wt% in Figure 1A) show a yielding behavior with a power law slope of −0.95 at $\dot{\gamma }$ < 0.5 s^−1^, and a strong shear thinning behavior at high shear rates. Storage modulus (G′) at ϕ_PVDF_ = 0 wt% in Figure 1B exhibits a plateau and is much larger than loss modulus (G″) in the whole range of ω, indicating a solid-like character of the CB network. When PVDF is added, the viscosity increases in the whole range of shear rates, while retaining power law slope at ϕ_PVDF_ = 2.5 wt%. G′ in Figure 1B is independent of the addition of PVDF, with G″ increase at high ω, which indicates that the addition of PVDF mainly increases matrix viscosity with a limited influence on the solid-like character of the CB network. 

**Figure 2:**
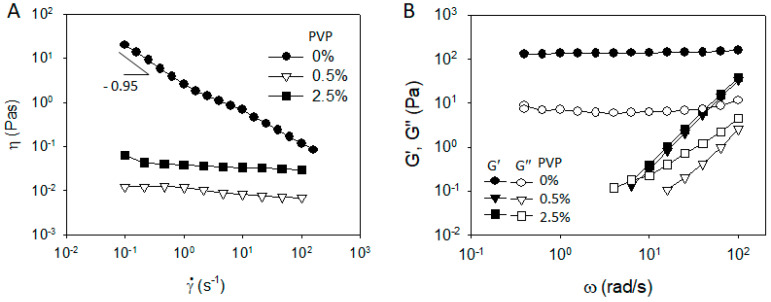
(**A**) Steady shear viscosity and (**B**) frequency dependent storage and loss modulus of CB/Polyvinylpyrrolidone (PVP)/NMP mixtures of 3 wt% (=1.3 vol%) of CB with a varying polymer concentration.

To highlight the role of PVDF in the rheological properties of the CB/PVDF/NMP suspension, we prepared the CB/PVP/NMP suspensions, where PVDF is replaced with PVP, and measured the rheological properties, as shown in Figure 2. In contrast to the case of CB/PVDF/NMP suspension (Figure 1A), steady shear viscosity of CB/PVP/NMP (Figure 2A) exhibits a Newtonian behavior at ϕ_PVP_ = 0.5 wt%, indicating a significant change of microstructures with the addition of PVP. G′ of CB/PVP/NMP suspension at ϕ_PVP_ = 0.5 wt% (Figure 2B) shows a significant relaxation with G′ << G″, indicating a structural transition from solid-like to liquid-like character of CB network with the addition of PVP. With a further addition of PVP from ϕ_PVP_ = 0.5 wt% to 2.5 wt%, the viscosity increases in the whole range of shear rates, maintaining Newtonian behavior (Figure 2A) and G″ increases with an identical value of G′ (Figure 2B), suggesting the increase in matrix viscosity without altering the microstructure of the CB network.

**Figure 3:**
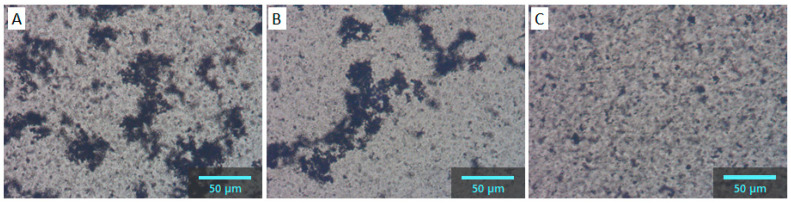
Optical microscopic images of (**A**) CB/NMP, (**B**) CB/PVDF/NMP (**C**) CB/PVP/NMP mixtures with 0.1 wt% of CB and 0.08 wt% of polymers.

To understand the difference in rheological properties of CB/PVDF/NMP (Figure 1) and CB/PVP/NMP (Figure 2), we observed a CB structure of the two suspensions with an optical microscope, as shown in Figure 3. The suspensions were diluted to 0.1 wt% (0.04 vol%) for microscopic observation. The aggregated structure of CB particles is observed in the absence of polymers (Figure 3A). Aggregated CBs are also observed in CB/PVDF/NMP suspension (Figure 3B), suggesting that the presence of PVDF does not stabilize CB particles, which supports the rheology data in Figure 1. On the contrary, CB aggregates were not observed in CB/PVP/NMP suspension (Figure 3C), suggesting a stabilization of CB particles with the addition of PVP. This shows a good consistency with the rheological results of CB/PVP/NMP (Figure 2). 

**Figure 4:**
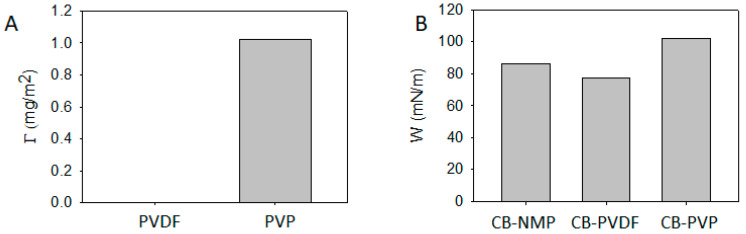
(**A**) Adsorption amount of PVDF and PVP on CB surface, respectively in NMP solvent. (**B**) Work of adhesion for CB-NMP, CB-PVDF and CB-PVP which suggests that PVP is preferably adsorbed on CB surface, whereas PVDF is not.

Now we investigate the origin of the structural formation of CB in the CB/PVDF/NMP and CB/PVP/NMP suspensions. Because the network structure of CBs is readily formed in suspension due to a strong van der Waals attraction, polymers are frequently introduced to disperse CB by adsorption on a CB surface, which sterically stabilizes the CB particles [25,31]. On this basis, we evaluated the adsorption properties of PVDF and PVP on the CB surface. Figure 4A exhibits the adsorption amount (Γ) of PVDF and PVP, respectively at polymer/CB ratio = 0.2. Γ of PVDF was measured to be nearly zero, implying a poor interaction between PVDF and CB. Non-adsorbing behavior of PVDF on CB can explain the nearly unchanged G′ (Figure 1) with the appearance of CB aggregates (Figure 3B) in the PVDF/NMP matrix, implying that PVDF increases matrix viscosity only with a negligible effect on the microstructure of CB particles. On the other hand, Γ of PVP is measured to be 1.02 mg/m^2^, suggesting there is a substantial interaction between PVP and CB. The adsorption of PVP explains a reduction of G′ (Figure 2) with a disappearance of CB aggregates (Figure 3C), indicating that PVP sterically stabilizes the CB particles by adsorption.

To understand the origin of the difference of adsorption behavior between PVDF and PVP onto CB surface, and to characterize the adsorption properties of two polymers in a more quantitative way, we compare the CB-PVDF interaction and CB-PVP interaction by calculating the work of adhesion [32]. We employed the Fowkes equation [32] to calculate the work of adhesion, which is expressed by Equation (1),

(1) $$W_{1-2}=2{\left(\gamma_{1}^{d}\gamma_{2}^{d}\right)}^{0.5}+2{\left(\gamma_{1}^{p}\gamma_{2}^{p}\right)}^{0.5}$$

where *W*_1–2_ is the work of adhesion of material 1 when it contacts material 2. *γ^d^* and *γ^p^* are the dispersive and polar part of surface energy, respectively. Subscripts 1 and 2 indicates materials 1 and 2. Dispersive and polar part of CB, PVDF, PVP and NMP are summarized in Table 1.

The work of adhesion for PVDF to CB is lower than NMP to CB (*W*_PVDF-CB_ < *W*_NMP-CB_), as in Figure 4B. This suggests that the affinity between PVDF and CB is poorer than NMP and CB, which explains why PVDF shows a nonadsorbing behavior on CB surface (Figure 4A) in CB/PVDF/NMP suspension. On the other hand, the work of adhesion for PVP to CB is higher than NMP to CB (*W*_PVP-CB_ > *W*_NMP-CB,_ Figure 4B), which implies that the affinity between PVP and CB is better than NMP and CB, which explains why PVP shows an adsorbing behavior on the CB surface (Figure 4A) in CB/PVP/NMP suspension. 

### 3.2. NCM/Polymer/NMP Mixtures

**Figure 5:**
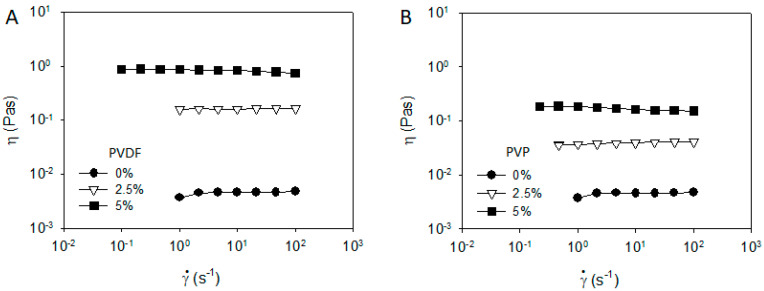
Steady shear viscosity of (**A**) Li(Ni_1/3_Mn_1/3_Co_1/3_)O_2_ (NCM)/PVDF/NMP and (**B**) NCM/PVP/NMP mixtures of 60wt% (=25 vol%) of NCM and 2.5 wt% polymer.

**Figure 6:**
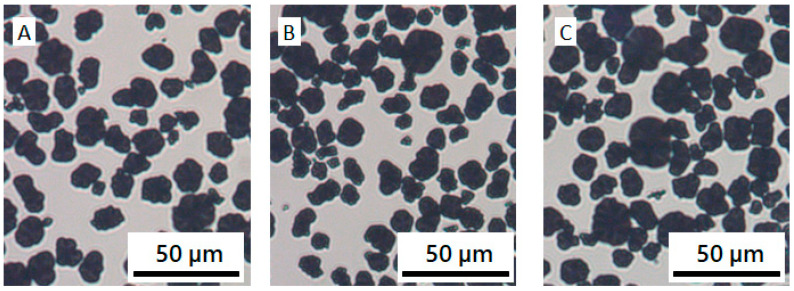
Microscope images of NCM particles in (**A**) NMP, (**B**) PVDF/NMP solution ϕ_PVDF_ = 2.5 wt%, and (**C**) PVP/NMP solution of ϕ_PVP_ = 2.5 wt%.

In this section, we characterized the rheology and microstructure of NCM/PVDF/NMP and NCM/PVP/NMP suspensions. A steady shear viscosity of NCM/PVDF/NMP in Figure 5A exhibits a Newtonian behavior at ϕ_PVDF_ = 0 wt%, implying that NCM particles do not exhibit a considerable aggregated structure. At a higher ϕ_PVDF_, the viscosity still shows a Newtonian behavior, although the viscosity increased more than an order of magnitude. This means that the addition of PVDF does not affect the dispersion quality of NCM particles, but only increases the matrix viscosity. The Newtonian behavior of NCM/PVDF/NMP suspension is supported by the microscopic images of NCMs in NMP in the absence (Figure 6A) and presence of PVDF (Figure 6B), which do not display aggregates for both cases. The viscosity of NCM/PVP/NMP suspensions in Figure 5B exhibit a similar behavior with NCM/PVDF/NMP (i.e., Newtonian behavior which is independent of polymer concentration), indicating that PVP does not affect dispersion of NCM particles. The Newtonian behavior of NCM/PVP/NMP suspension is supported by the microscope images of NCM in NMP in the absence (Figure 6A) and presence of PVP (Figure 6C), which do not exhibit a considerable aggregated NCMs for both cases. The limited effect of both PVP and PVDF on NCM was supported by the measurement of polymer adsorption on NCM, resulting in a negligible amount of Γ for both PVDF and PVP (result not shown). 

### 3.3. NCM/CB/Polymer/NMP Suspensions

In this section, we compared the rheological properties and optical microscope images of NCM/CB/PVDF/NMP and NCM/CB/PVP/NMP suspensions, which include all the constituents of actual cathode LIB slurries. 

**Figure 7:**
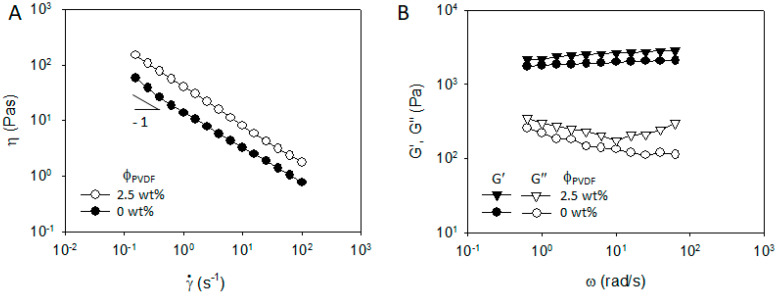
(**A**) Steady shear viscosity and (**B**) frequency dependent storage and loss modulus of NCM/CB/PVDF/NMP mixtures of 60 wt% (=25 vol%) NCM and 1.5 wt% (=1.3 vol%) CB in PVDF/NMP solution at ϕ_PVDF_ = 0 wt% and 2.5 wt%.

The steady shear viscosity of NCM/CB/PVDF/NMP suspensions in Figure 7A exhibits a typical yielding behavior with a power law slope of −1 at $\dot{\gamma }$ < 0.5 s^−1^, and a strong shear thinning behavior with the increase in shear rate at ϕ_PVDF_ = 0 wt%. The viscosity at ϕ_PVDF_ = 2.5 wt% shows an identical behavior with ϕ_PVDF_ = 0 wt%, implying that PVDF hardly affects flow-induced structural response in NCM/CB/PVDF/NMP suspension. Frequency dependent storage modulus (G′) at ϕ_PVDF_ = 0 wt% in Figure 7B exhibits a plateau value and is much larger than loss modulus (G″) in the whole range of ω, indicating a solid-like character of the structure formed from NCM and CB particles. When PVDF is added, G′ and G″ slightly increases in the whole frequency range, with an upturn of G″ at high ω indicating an increasing matrix viscosity in the suspension. The evolution of G′ and G″ with the addition of PVDF implies that, at least, PVDF does not weaken the solid-like character of NCM and CB structure.

**Figure 8:**
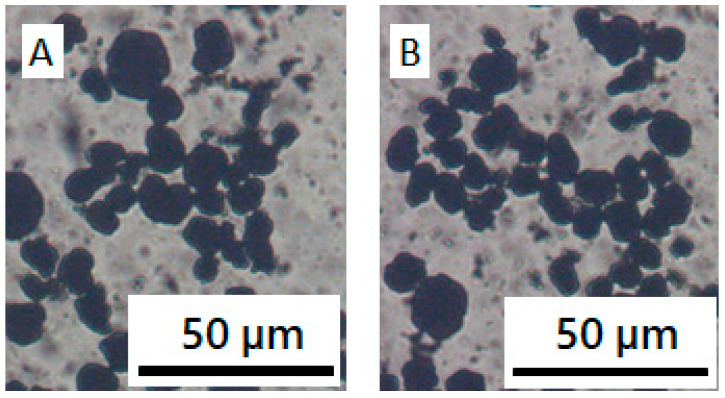
Microscope images of NCM and CB in PVDF/NMP solution with a varying ϕ_PVDF_. (**A**) 0 wt%, (**B**) 2.5 wt%.

To understand the structure of CBs and NCMs in NCM/CB/PVDF/NMP suspension, we diluted the suspension and observed its structure by means of an optical microscope. Figure 8A shows the aggregated structure of NCM and CBs, supporting the solid-like character in rheological measurement. Aggregates of NCM and CB were independent of the presence of PVDF, as shown in Figure 8B, implying that PVDF does not stabilize the structure of NCM and CB structure, which is again consistent with the rheological measurement (Figure 7). It needs to be noted that the mixture of NCM and CBs form an aggregated structure, whereas NCM particles are moderately dispersed in NMP in Figure 8A. This implies there is an attraction between CB and NCM in cathode slurry. A clear understanding of the interaction between CB and NCM, and the structure formed from CB and NCM in the cathode slurries should be a focus of study for future work.

**Figure 9:**
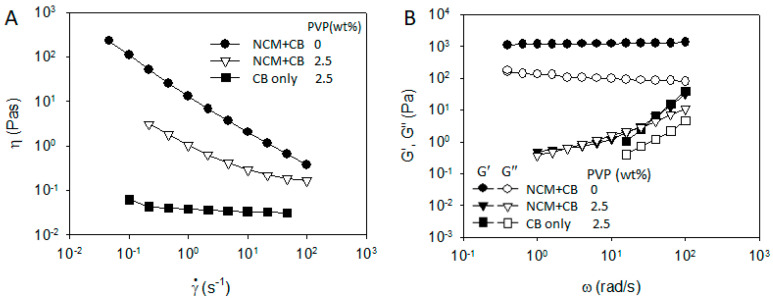
(**A**) Steady shear viscosity and (**B**) frequency dependent storage and loss modulus of NCM/CB/PVP/NMP mixtures of 60 wt% (=25 vol%) NCM and 1.5 wt% (=1.3 vol%) CB in PVDF/NMP solution at ϕ_PVDF_ = 2.5 wt%.

**Figure 10:**
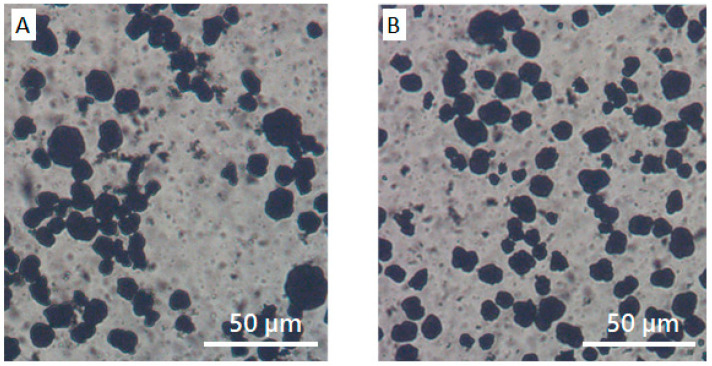
Microscope images of (**A**) NCM/CB/NMP and (**B**) NCM/CB/PVP/NMP.

In contrast to the role of PVDF in the rheology and microstructure of NCM/CB/ PVDF/NMP suspension, PVP plays a remarkably different role in NCM/CB/PVP/NMP suspension. Compared with the steady shear viscosity of NCM/CB/PVP/NMP suspension at ϕ_PVP_ = 0 wt%, the viscosity of the suspension at ϕ_PVP_ = 2.5 wt% is significantly reduced in the whole range of shear rate, with an occurrence of Newtonian plateau at high shear rate, as in Figure 9A. Frequency dependent linear viscoelastic property in Figure 9B shows a significant transition with an addition of PVP from solid-like character of G′ and G″ (plateau G’ at low ω with G′ >> G″) at ϕ_PVP_ = 0 wt% to liquid-like character at ϕ_PVP_ = 2.5 wt% (relaxation of G’ with G′ < G″). The transition of rheological properties with the addition of PVP may suggest a breakup of network structure of NCM and CB which was previously observed in a microscopic image (Figure 8A). To check the effect of PVP on the aggregated structure of NCM and CB, we obtained optical microscopic images of NCM/CB/PVP/NMP in the absence and presence of PVP. Aggregated structure of NCM and CB at ϕ_PVP_ = 0 wt% (Figure 10A) became considerably dispersed at ϕ_PVP_ = 2.5 wt% (Figure 10B). This suggests that PVP stabilizes the aggregated structure of CB and NCM. The stabilization of NCM and CB by PVP can be explained by the adsorbing behavior of PVP on the CB surface, as described in Figure 4A.

## 4. Conclusions

In the present study, we investigated the role of Poly(vinylidene fluoride) (PVDF) on the rheology of cathode battery slurries consisting of Li(Ni_1/3_Mn_1/3_Co_1/3_)O_2_ (NCM), carbon black (CB) and N-Methyl-2-pyrrolidone (NMP). The role of PVDF could be understood with the following three model suspensions: CB/polymer/NMP, NCM/Polymer/NMP, and NCM/CB/Polymer/NMP. To further understand the role of PVDF, we selected PVP and compared the rheology and microstructure of PVDF-based and PVP-based model mixtures. We found that PVDF adsorbed neither onto the NCM nor the CB surface, which can be attributed to its poor affinity to NCM or CB. Rheological study suggested that PVDF mainly increased matrix viscosity in CB/PVDF/NMP mixture, almost without affecting the network structure of CB particles. Furthermore, we observed NCM-CB networks in NCM/CB/PVDF/NMP mixture, where PVDF hardly affected the NCM-CB structure. On the other hand, PVP adsorbed on CB surface, thus stabilizing CB particles in the CB/PVP/NMP mixture. Furthermore, NCM-CB networks in the NCM/CB/PVP/NMP mixture were stabilized with the addition of PVP. 

Since it has been found that PVDF and PVP play distinctly different rolea in rheological property and dispersion stability in the cathode slurry, we expect their roles will be reflected in the microstructure and electrochemical performances of the dried electrode composite [14]. Although understanding the role of these binders in a dry electrode is of great importance, this is outside the scope of our current study, and will be further investigated in future work.

## Figures and Tables

**Table 1:** Dispersive and polar part of surface energy of CB, NMP, PVDF and PVP.

	CB [32]	NMP [33]	PVDF [32]	PVP [34]
Dispersive part, *γ^d^*	56.27	29.21	24.33	43.4
Polar part, *γ^p^*	0.54	11.58	6.18	5.1
